# Early Functional and Morphological Muscle Adaptations During Short-Term Inertial-Squat Training

**DOI:** 10.3389/fphys.2018.01265

**Published:** 2018-09-10

**Authors:** Víctor Illera-Domínguez, Sergi Nuell, Gerard Carmona, Josep M. Padullés, Xavier Padullés, Mario Lloret, Roser Cussó, Xavier Alomar, Joan A. Cadefau

**Affiliations:** ^1^Institut Nacional d’Educació Física de Catalunya (INEFC), Universitat de Barcelona, Barcelona, Spain; ^2^Tecnocampus, Escola Superior de Ciències de la Salut, Universitat Pompeu Fabra, Mataró, Spain; ^3^Departament de Biomedicina, Universitat de Barcelona, Barcelona, Spain; ^4^Creu Blanca, Barcelona, Spain

**Keywords:** flywheel, strength, resistance training, hypertrophy, MRI

## Abstract

**Purpose:** To assess early changes in muscle function and hypertrophy, measured as increases in muscle cross-sectional areas (CSAs) and total volume, over a 4 weeks inertial resistance training (RT) program.

**Methods:** Ten young RT-naive volunteers (age 23.4 ± 4.1 years) underwent 10 training sessions (2–3 per week) consisting of five sets of 10 flywheel squats (moment of inertia 900 kg⋅cm^2^). Magnetic resonance imaging (MRI) scans of both thighs were performed before (PRE), and after 2 (IN) and 4 (POST) weeks of training to compute individual muscle volumes and regional CSAs. Scans were performed after ≥96 h of recovery after training sessions, to avoid any influence of acute muscle swelling. PRE and POST regional muscle activation was assessed using muscle functional MRI (mfMRI) scans. Concentric (CON) and eccentric (ECC) squat force and power, as well as maximal voluntary isometric contraction force (MVIC) of knee extensors and flexors, were measured in every training session.

**Results:** Significant quadriceps hypertrophy was detected during (IN: 5.5% ± 1.9%) and after (POST: 8.6% ± 3.6%) the training program. Increases in squat force (CON: 32% ± 15%, ECC: 31 ± 15%) and power (CON: 51% ± 30%, ECC: 48% ± 27%) were observed over the training program. Knee extensor MVIC significantly increased 28% ± 17% after training, but no changes were seen in knee flexor MVIC. No correlation was found between regional muscular activation in the first session and the % of increase in regional CSAs (*r* = -0.043, *P* = 0.164).

**Conclusion:** This study reports the earliest onset of whole-muscle hypertrophy documented to date. The process initiates early and continues in response to RT, contributing to initial increases in force. The results call into question the reliability of mfMRI as a tool for predicting the potential hypertrophic effects of a given strengthening exercise.

## Introduction

Resistance training (RT) has been shown to induce profound and specific changes in virtually all biological systems (Egan and Zierath, 2013). Optimizing the stimulus and time invested to produce these changes is one of the goals of strength and conditioning trainers. In an attempt to achieve this, RT programs are usually periodized, based on the different dynamics of adaptation for each physiological variable. Muscle hypertrophy induced by RT has generally been considered to be a slow process with a delayed onset, with initial strength gains mostly attributed to neural factors (Moritani and DeVries, 1979; Blazevich et al., 2007). Biologically, muscle hypertrophy is the result of a positive balance between the synthesis and breakdown of proteins, which is manifested as microscopic (fiber thickness) and macroscopic (muscle thickness) surrogate variables. It is known that remodeling processes are highly dynamic in skeletal muscle, and that single bouts of RT immediately upregulate intramuscular anabolic signaling, amino acid transport and protein synthesis (Bickel et al., 2005; Gonzalez et al., 2016; Song et al., 2017). Therefore, if the protein balance remains positive after RT sessions (Reitelseder et al., 2014), it would be theoretically possible to accrete muscle mass early, starting after the very first session. In line with this idea, in a recent clinical trial in humans, increases in Type II fiber cross-sectional area (CSA) were detected after only 2 weeks of RT (Holloway et al., 2017). Although this histological evidence suggests a continuous process of adaptation, the macroscopic evidence of adaptation in the early phase of RT is less convincing.

Given this background, the time course of macroscopic muscle hypertrophy was revisited in a recent review of studies involving different RT protocols and at least three muscle size measurements over time (Counts et al., 2017). Even though most of the studies analyzed used a similar sample (untrained young volunteers), a lack of agreement between the results was observed. For the lower limbs, the reported results range from no significant changes in muscle size after 5–12 weeks (Abe et al., 2000; Blazevich et al., 2007) to significant muscle hypertrophy after just 3–4 weeks of RT (Lüthi et al., 1986; Narici et al., 1989; Kubo et al., 2010; Baroni et al., 2013; Brook et al., 2015). To date, the study conducted by Seynnes et al. (2007) reports the earliest onset of macroscopic muscle hypertrophy, showing an increase of quadriceps femoris (QUAD) CSA after only 20 days of training (nine training sessions). The differences in the training stimulus, frequency of the assessments and sensitivity of the measurement methods all contribute to the spread of results in the available literature (Counts et al., 2017). It is hoped that new studies using an optimized RT stimulus, and more sensitive and frequent assessment will shed light on the topic.

**FIGURE 1:**
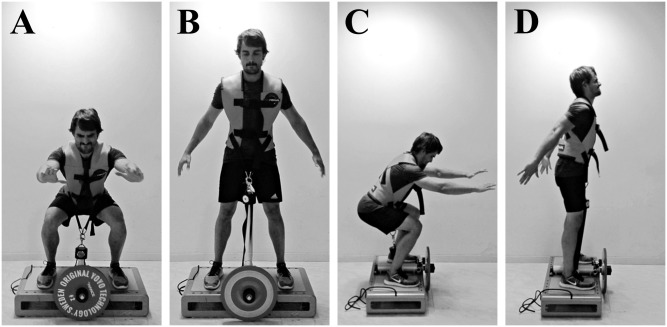
Concentric–eccentric bilateral half-squats performed on a “YoYo squat” inertial flywheel device; anterior view **(A,B)**, lateral view **(C,D)**. **(A,C)** Bottom position of the exercise and transition between the eccentric and the concentric phases. **(B,D)** Top position of the exercise and transition between the concentric and the eccentric phases. The vest is attached to a strap wound on the flywheel axis. As resistance is the moment of inertia of the flywheel, the force applied during the concentric phase to unwind the strap determines the force needed during the eccentric phase (as the strap rewinds) to impede the spin of the axis.

In order to produce fast and significant increases in muscle size, the training stimulus has to meet certain characteristics. Mechanical tension is the primary driver of muscle hypertrophy (Schoenfeld, 2010). Thus, RT interventions aiming to increase muscle volume should focus on the magnitude and the length of time producing tension. Several training systems and techniques can be used to achieve an enhanced stimulus. For instance, inertial flywheels allow for accommodated maximal or near maximal actions from the very first repetition of a set in the concentric (CON) and eccentric (ECC) phases (Tesch et al., 2004; see **Figure 1**). To the best of our knowledge, Lundberg et al. (2013) reported the fastest rate of whole muscle hypertrophy over a period of 5 weeks (0.4% increase per day), using a combination of flywheel RT and intense aerobic exercise. In a recent meta-analysis, flywheel training showed to be more effective than conventional weights in promoting increases in muscle volume, strength and power (Maroto-Izquierdo et al., 2017).

A wide range of techniques can be employed to estimate changes in whole-muscle size (Counts et al., 2017; Franchi et al., 2018). Magnetic resonance imaging (MRI) is regarded as the gold standard for clinical and research imaging of skeletal muscle, and is commonly used to compute 1 (middle), 2 (proximal and distal), or 3 (proximal, middle and distal) CSAs of a given muscle (Seynnes et al., 2007). However, single CSAs may not be representative of whole-muscle changes, given that different patterns of hypertrophy (ventral or distal) have been reported in response to specific CON or ECC loading (Franchi et al., 2017). New MRI approaches for the assessment of total muscle volume, and not only CSAs of specific muscle sites, provide a better measurement of whole-muscle changes (Nordez et al., 2009).

Other acute physiological processes need to be carefully taken into account when measuring muscle hypertrophy. RT results in a rapid activity-dependent influx of fluid and accumulation of osmolytes (phosphate, lactate, and sodium) and a subsequent acute inflammatory response that can last for several hours after exercise (Damas et al., 2016). These processes temporally alter muscle volume and may interfere with hypertrophy measurements (DeFreitas et al., 2011), but they can also provide valuable information. For instance, acute fluid changes in muscles can be assessed indirectly by muscle functional MRI (mfMRI) (Damon et al., 2007). This non-invasive technique measures the “T2 shift,” which is the increase in the transverse relaxation time (T2) of muscle water from pre-exercise to post-exercise (Cagnie et al., 2011). The T2 shift is positively correlated with electromyographic activity, at least when muscle groups are exercised in isolation (Adams et al., 1992). In consequence, and although it can only be considered as a proxy marker, mfMRI is commonly used as a tool to determine total or regional muscle metabolic activation in different exercises (Fernandez-Gonzalo et al., 2016). Given that swelling is caused by muscular activity, and that cell swelling is a known upregulator of anabolic signaling pathways in a wide variety of cells (Lang, 2007), it would be reasonable to expect that muscles with greater T2 shifts after acute RT will display greater hypertrophy if the same exercise is systematically repeated over time. In fact, previous studies have found a correlation between these parameters when an isolated muscle or muscle group is exercised over time (Wakahara et al., 2012, 2013).

The purpose of this study was to assess early changes in thigh muscles function (force and power), hypertrophy (total volume and regional CSAs), and muscle activation (T2 shift), during a 4 weeks inertial-squat RT program. We hypothesized that inertial RT would produce early (≈2 weeks) detectable increases in muscle function and size. Additionally, we studied the relationship between muscle hypertrophy and activation in the first session, under the hypothesis that different degrees of hypertrophy of thigh muscles would be directly related to muscle task-specific activity assessed by mfMRI.

## Materials and Methods

### Experimental Approach

**FIGURE 2:**
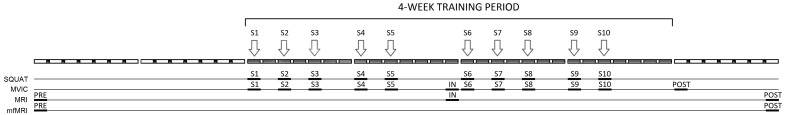
Schematic representation of the study protocol. S1–S10 training sessions, SQUAT squat performance (kinetics), MVIC maximal voluntary isometric contraction force of knee extensors and knee flexors, MRI muscle volume assessed by magnetic resonance imaging, mfMRI muscle activation assessed by muscle functional magnetic resonance imaging.

A quasi-experimental design of repeated measurements was used to determine the effects of an intervention on different variables. The independent variable in this study was a 4 weeks training program consisting of bilateral half-squats performed on an inertial flywheel device. The variables assessed for thigh muscles were: muscular activation, muscle hypertrophy and muscle performance. Activation was assessed through the T2 shift by mfMRI. Hypertrophy was assessed through increases in total volume and regional CSAs using MRI. Muscle performance was assessed through maximal voluntary isometric contraction force (MVIC) of the knee extensors and knee flexors, as well as by squat dynamic force and power. The assessments were performed before (PRE), and after 2 (IN) and 4 (POST) weeks of training; and also during each of the 10 training sessions. **Figure 2** is a schematic representation of the study protocol.

### Subjects

Ten young volunteers (men = 8; women = 2) (age 23.4 ± 4.1 years; height 174.8 ± 7.7 cm; weight 71.0 ± 6.8 kg; self-reported weekly moderate intensity activity 6.3 ± 3.4 h⋅week^-1^) who had not suffered muscle or tendon injuries in the previous 6 months were recruited for the study. All were recreationally active physical education students who had not been involved in any RT program during the 6 months preceding the study. The experiment was conducted in accordance with the code of ethics of the World Medical Association (Declaration of Helsinki) and was approved by the Ethics Committee of the Catalan Sports Council (Generalitat de Catalunya). A familiarization session was conducted 2 weeks before starting the study. All the volunteers were informed of the aims, experimental protocol, procedures, benefits, and risks of the study, and their written informed consent was obtained. The volunteers were instructed to maintain their usual level of physical activity throughout the experimental period, and to refrain from moderate or heavy physical activities in the 96 h before the MRI.

### Procedures

#### Training Protocols

The training period comprised of 10 training sessions distributed over 4 weeks (two or three sessions per week). Each training session consisted of a standardized warm-up (two sets of 10 body-weight squats, with 1 min of rest between the sets), followed by five sets of flywheel exercise. Each set of exercise included three sub-maximal repetitions to accelerate the disk, followed by 10 maximal voluntary repetitions. There was 3 min of rest between sets. Exercise consisted of CON – ECC bilateral half-squats performed on a “YoYo squat” inertial flywheel device (YoYo Technology AB, Stockholm, Sweden) (**Figure 1**). Given the properties of the flywheel, the resulting moment of inertia was 900 kg⋅cm^2^. The start and end position of each repetition was a 90° knee angle. The participants were verbally encouraged to perform the concentric phase at their fastest voluntary speed.

#### Squat Performance

Exercise kinetics were monitored during each session using a Chronopic friction encoder (Chronojump, Barcelona, Spain), (accuracy ± 1 mm, sampling rate 1000 Hz). The sensor was tightly attached at a known diameter of the flywheel, sharing the same linear speed. The set-up parameters such as disk inertia, axis diameter, and the volunteer’s body mass were entered into the associated Chronojump software (v1.6.0.0) to compute the kinematic exercise variables in real time. Visual and acoustic feedback on the performance was provided for each repetition using a computer. The variables calculated for the CON and ECC phase of each repetition were displacement and mean values of force and power. Chronojump is open-code software, and a complete repository of the code and formulas used can be found online (Chronojump, 2018).

#### MVIC

Maximal voluntary force production of the knee extensors and knee flexors was tested in isometric conditions. For data collection, a strain gauge and a custom-built bench were used. The signal was recorded at a frequency of 200 Hz, using a Muscle Lab 6000 (Ergotest Technology AS, Porsgrunn, Norway) and real-time results were provided on a computer monitor. For assessment of knee extensors, the volunteers were positioned supine (knee angle = 90°; hip angle = 180°); for knee flexors, lying prone (knee angle = 135°; hip angle = 180°). In both positions, the volunteers were fixed using adjustable straps and the strain gauge was attached at the mid-level of the malleolus tibiae forming a perpendicular angle with the leg. Three unilateral 5 s trials were recorded for the dominant limb, with 30 s of recovery between trials. Subjects were instructed and verbally encouraged to perform and maintain maximum force output. Pre-contraction conditions were standardized and attempts with counter movements were rejected. Maximum force values were selected using mean force over a 1 s mobile window once a force plateau had been established (Tesch et al., 2004; Carmona et al., 2018). The best attempt was selected at each time point for further analysis. MVIC was assessed before each training session, and at IN and POST, after the standard warm-up.

#### Image Acquisition and Processing

MRI was used to compute the volumes of the individual muscles of the thigh and mfMRI to assess the acute muscle activation after exercise. The volunteers were placed supine inside a 3-T MRI scanner (Magnetom VERIO, Siemens, Erlangen, Germany), with their heads outside the MR-bore and thighs covered with one 32- and two flexible 4-channel coils, respectively, in the proximal and distal segments. A custom-made foot-restraint device was used to standardize and fix limb position, and to avoid any compression of thigh muscles. To ensure the same anatomical area was assessed each time, the range was centered at the mid-length of the femur, as measured on the coronal plane image.

##### Muscle activation

For the assessment of PRE and POST muscle activation, one scan was performed in basal conditions and another 3–5 min after finishing an acute bout of the exercise (Cagnie et al., 2011; Mendez-Villanueva et al., 2016) (10 sets of 10 flywheel squats). To minimize the effects of fluid shifts caused by walking, the volunteers remained recumbent for a minimum of 10 min before basal data acquisition (LeBlanc et al., 2000), and were assisted over the 10 m between the exercise room and the MRI scan. In each scan, 12 contiguous (each 31.5 mm) 3.5 mm cross-sectional images of both thighs were obtained using the following scan sequence: SE MULTIECO, repetition time: 1800 ms, echo times: 20, 40, 60, 80, and 100 ms, field of view: 40 × 40 cm, matrix: 224 × 320, and total acquisition time: 6 min. A parametric image was generated from the T2 mapping sequence using Leonardo workstation (Siemens). The T2 of the muscles in both thighs was measured using OsiriX 8.5.2. (Pixmeo, Geneva, Switzerland). The assessment was performed by the same researcher on all occasions. All the sequences from a given volunteer were processed in parallel, with the researcher blinded. A circular region of interest (ROI) was selected for the muscles gluteus maximus (GM), rectus femoris (RF), vastus intermedius (VI), vastus medialis (VM), vastus lateralis (VL), adductor magnus (AM), gracilis (GR), biceps femoris long head (BFL), biceps femoris short head (BFS), semitendinosus (ST), and semimembranosus (SM) in each of the T2 mapping images where these muscles were visible. ROIs of similar size and anatomical location were placed in the subsequent image sets to ensure positioning identical to that in the first analysis (Mendez-Villanueva et al., 2016). The intra-class correlation coefficients and coefficient of variation for the intra-rater agreement of the T2 values were: 0.97 ± 0.06 and 1.09% ± 0.54% for the different muscles assessed. The T2 values for each muscle were computed as the mean value of the different ROIs. The T2 shift was then calculated by subtracting T2 basal values from T2 values post-exercise, and expressed as a percentage of the basal value. The results are shown as the average T2 shift of right and left thighs.

##### Muscle volume

**FIGURE 3:**
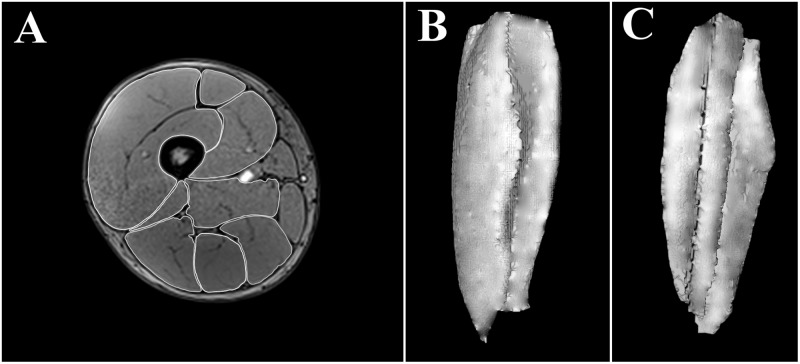
Manually outlined muscle perimeters in a single cross-sectional image (≈50% length) of a right thigh **(A)**. Three-dimensional reconstruction of a right vastus intermedius + vastus lateralis from all (≈250) the cross sectional areas assessed; anterior view **(B)**, posterior view **(C)**.

To assess muscle volume, PRE, IN, and POST scans were performed. To minimize the effects of fluid shifts caused by walking (LeBlanc et al., 2000), the volunteers remained recumbent for a minimum of 10 min before data acquisition. In each scan, 288 contiguous (each 1.5 mm) 1.5 mm cross-sectional images of both thighs were obtained, using the following scan sequence: VIBE 3D Dixon, field of view: 40 cm × 40 cm, matrix: 320 × 320, and total acquisition time: 2 min. The edges of the RF, VM, VL+VI, adductors (ADD), BFL, BFS, ST, and SM muscles were manually outlined, image by image, by the same researcher using OsiriX 8.5.2. (Pixmeo, Geneva, Switzerland) (**Figure 3**). Because there may appear to be substantial fusion between VL and VI on some slices (Pareja-Blanco et al., 2016), these muscles were outlined together (VL+VI). The same approach was adopted for the assessment of ADD volume, which includes pectineus, and adductor longus, brevis and magnus. All the sequences from a given volunteer were processed in parallel, with the researcher blinded. The total volume of muscles was computed from all the CSAs of the images where they were visible, in the range within the last image where the ischial tuberosity was visible and the last image where the femoral condyles were visible (**Figure 3**). The intra-class correlation coefficients and coefficient of variation for the intra-rater agreement of the CSA segmentation were 1.00 ± 0.00 and 1.44% ± 0.85% for the different muscles assessed. The intra-class correlation coefficients and coefficient of variation for the intra-rater agreement of the volumetric values were 1.00 ± 0.00 and 0.57% ± 0.42% for the different muscles assessed, similar to previous estimations of error using this method (Nordez et al., 2009). QUAD, hamstrings (HAMS) and total thigh muscle volume were computed *post hoc* as follows:

QUAD=RF+VM+VL+VI

HAMS=BFL+BFS+ST+SM

TOTAL=QUAD+HAMS+ADD

Volume changes were calculated by subtracting the PRE muscle volume from that IN or POST, expressed as a percentage of the PRE value. The results are shown as the average of right and left thighs.

As depicted in **Figure 2**, the scans were performed with at least 96 h of recovery after the previous training session, so as to account only for hypertrophic changes and avoid any influence of acute muscle swelling (Damas et al., 2016). To verify the absence of muscle edema at IN and POST, basal T2 values of the assessed muscles were compared with those obtained at PRE (Damas et al., 2016).

##### Regional muscle activation and hypertrophy

Complementary analysis was performed to evaluate regional activation and hypertrophy of the muscles assessed and the relationship between these variables. PRE SE MULTIECO, and both PRE and POST VIBE 3D sequences were spatially synchronized. Thus, PRE regional T2 shifts were matched with PRE and POST regional CSAs. The exact anatomical placement of the cross-sectional images where T2 ROIs were measured was used to assess regional CSAs. Due to the differences in the protocols used for assessing these variables, the regional T2 shift of AM and the mean regional T2 shifts of VL and VI were matched with the ADD and VL+VI regional CSAs, respectively.

#### Statistical Analysis

Data are presented as mean ± SD. All statistical analysis was performed using SPSS v.23.0.0.0 (IBM, Armonk, NY, United States). A normal distribution of the data was checked using the Shapiro–Wilk test and Levene’s test for homogeneity of variances was performed at each time level. For normal data, one-way ANOVA for repeated measures (with Mauchly’s sphericity test) was used to determine the effects of time (PRE, sessions 1–5, IN, sessions 6–10, POST) on the different parameters assessed (see **Figure 2**). When significant effects were found, *post hoc* testing was performed by applying paired *t*-tests with a Bonferroni correction for multiple comparisons. For non-normal data, Friedman’s test was applied. For the assessment of within-subject squat displacement variability, coefficients of variation were calculated for all the squat repetitions performed by each volunteer. Differences between CON and ECC force and power were calculated using *t*-tests for paired samples. The correlation between regional T2 shifts and regional CSAs was assessed by Pearson’s product *r*-values. Due to differences in femur lengths and muscles anatomy between participants, only regional T2 shifts and CSAs of anatomical regions for which the sample was *n* > 7 volunteers appear in the results section. Statistical significance was set at *P* < 0.05.

## Results

### Squat Performance

**FIGURE 4:**
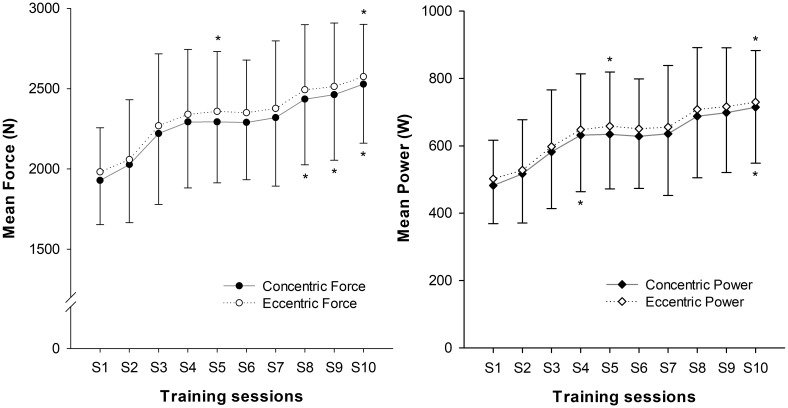
Progression of squat mean values of force and power over the 10 training sessions (S). (^∗^) indicates significant changes from baseline values (*P* < 0.05).

Mean displacement during the training sessions was 294 ± 24 mm, with low within-subject displacement variability (4.7% ± 1.5%). Higher forces (2.23% ± 0.39%; *P* = 0.008) and power (2.98% ± 0.69%; *P* = 0.011) were developed during the ECC than the CON phase. Significant effects of time were found for CON (*P* < 0.001) and ECC (*P* = 0.001) force, and for CON (*P* = 0.001) and ECC (*P* = 0.002) power. Increases in squat force (CON 32% ± 15%, ECC 31% ± 15%) and power (CON 51% ± 30%, ECC 48% ± 27%) were seen after the training period (**Figure 4**).

### MVIC

**FIGURE 5:**
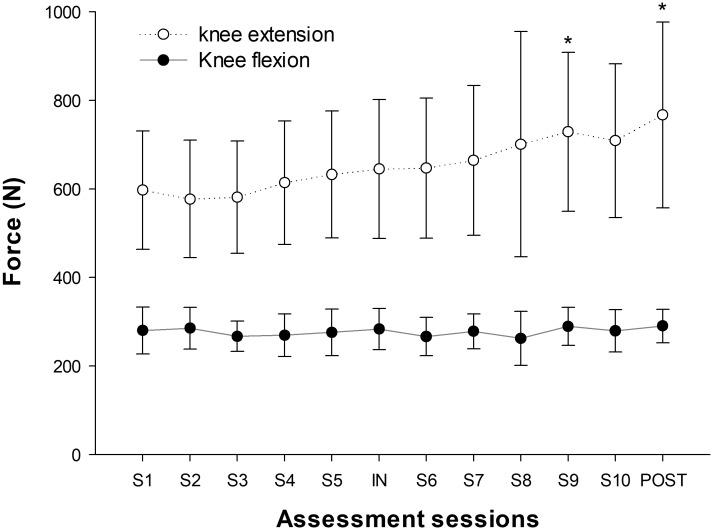
Progression of MVIC force values throughout the training period. (^∗^) indicates significant changes from baseline values (*P* < 0.05).

Significant increases in knee extensor MVIC were seen over the training sessions (*P* < 0.001), with an overall increase of 28% ± 17% after the training period. No significant changes were seen in knee flexor MVIC (*P* = 0.368) (**Figure 5**).

### Muscle Activation

**FIGURE 6:**
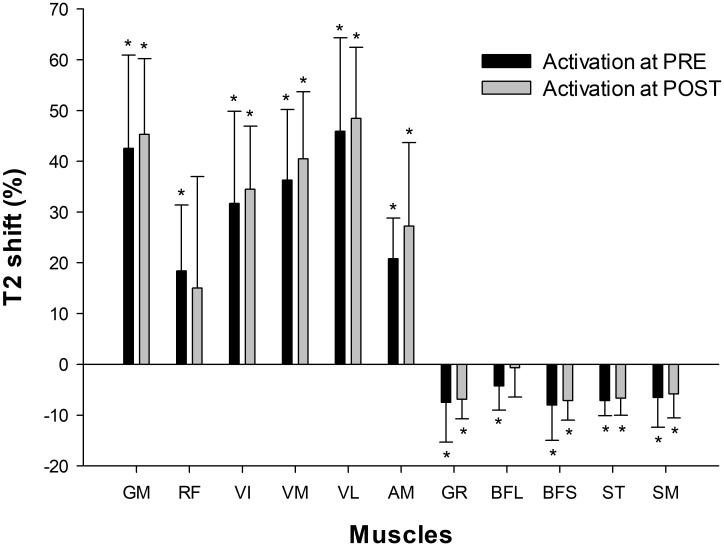
Muscular activation assessed by mfMRI (T2 shift) after 10 sets of 10 maximal squats on the flywheel device; before (PRE) and after (POST) the training period. GM, gluteus maximus; RF, rectus femoris; VI, vastus intermedius; VM, vastus medialis; VL, vastus lateralis; AM, adductor magnus; GR, gracilis; BFL, biceps femoris long head; BFS, biceps femoris short head; ST, semitendinosus; SM, semimembranosus. (^∗^) indicates significant post-exercise changes from basal T2 values (*P* < 0.05). No significant differences were found between PRE and POST activation.

The number of images analyzed per subject for each muscle was GM 3.4 ± 0.5; RF 7.3 ± 0.7; VI 8.4 ± 1.1; VM 8.7 ± 0.7; VL 9.4 ± 0.7; AM 7.0 ± 1.0; GR 8.8 ± 0.8; BFL 7.3 ± 0.7, BFS 5.2 ± 0.8, ST 8.3 ± 0.7, SM 6.4 ± 1.0. Of these, GM, RF, VI, VM, VL, and AM showed a positive T2 shift, whereas GR, BFL, BFS, ST, and SM showed a negative T2 shift (**Figure 6**). No significant effects of time (PRE vs. POST) were seen in T2 shift in any of the muscles assessed.

### Muscle Volume

**FIGURE 7:**
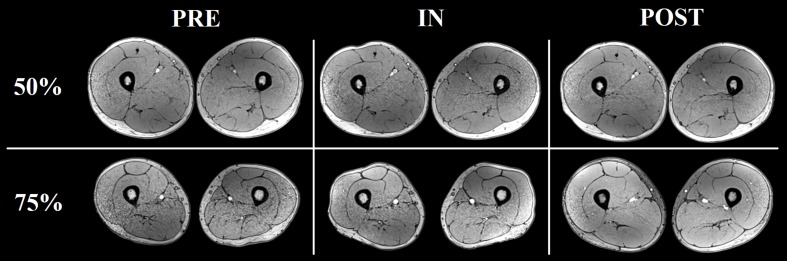
Representative increases in thigh muscles CSA (at ≈ 50% and ≈ 75% the length of the femur) as assessed by consecutive MRI scans before (PRE), and after 5 (IN) and 10 (POST) training sessions from one of the volunteers.

There was a significant main effect of time for all the muscles analyzed, except for SM (**Table 1**). Significant increases in RF, VM, VL+VI, ADD, and BFL volume were detected from PRE to IN and from PRE to POST (all *P* < 0.05). A representative case is shown in **Figure 7**.

**Table 1:** Muscle volumes at baseline (PRE) and after 5 (IN) and 10 training sessions (POST).

	Volume (cm^3^)			Volume change (%)		*P*-value time effect
			
	PRE	IN	POST	PRE-IN	PRE–POST	
Individual Muscles					

RF	237.0 ± 42.9	244.5 ± 45.2^∗^	248.1 ± 46.9^∗^	3.2^∗^	4.7^∗^	<0.001
VM	472.6 ± 107.6	501.5 ± 110.2^∗^	518.9 ± 125.3^∗^	6.1^∗^	9.8^∗^	<0.001
VI+VL	1250.1 ± 233.0	1320.8 ± 239.9^∗^	1360.7 ± 253.1^∗^	5.7^∗^	8.8^∗^	<0.001
BFL	224.9 ± 28.0	229.6 ± 30.5^∗^	232.8 ± 29.6^∗^	2.1^∗^	3.5^∗^	<0.001
BFS	105.0 ± 23.8	104.5 ± 23.5	108.4 ± 22.5	-0.5	3.2	0.028
ST	231.6 ± 45.2	232.9 ± 44.8	238.4 ± 47.5	0.6	3.0	0.020
SM	246.7 ± 31.0	246.3 ± 32.8	250.9 ± 33.7	-0.2	1.7	0.066

Muscle Groups					

QUAD	1959.8 ± 358.2	2066.8 ± 369.9^∗^	2127.7 ± 399.2^∗^	5.5^∗^	8.6^∗^	<0.001
ADD	1037.8 ± 141.5	1065.4 ± 147.1^∗^	1096.2 ± 172.7^∗^	2.7^∗^	5.6^∗^	<0.001
HAMS	808.2 ± 108.7	813.3 ± 109.7	830.5 ± 112.0^∗^	0.6	2.8^∗^	<0.001

Σ Thigh Muscles					

Total	3805.7 ± 583.3	3945.5 ± 600.1^∗^	4054.4 ± 656.8^∗^	3.7^∗^	6.5^∗^	<0.001


**RF, rectus femoris; VM, vastus medialis; VI+VL, vastus intermedius + vastus lateralis; BFL, biceps femoris long head; BFS, biceps femoris short head; ST, semitendinosus; SM, semimembranosus; QUAD, quadriceps; ADD, adductors; HAMS, hamstrings. (^∗^) indicates significant changes from basal values (P < 0.05).**

### Regional Muscle Activation and Hypertrophy

**FIGURE 8:**
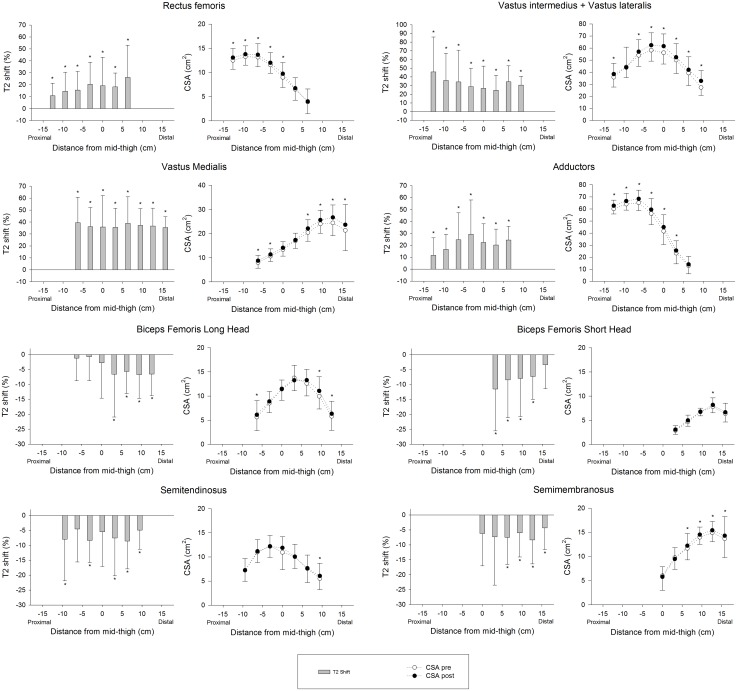
Regional muscle activation assessed as T2 shift in the first session (PRE) and changes in regional CSAs from baseline (PRE) to the end of the 4 weeks training intervention (POST). In the activation graphs (left), (^∗^) indicates significant post-exercise changes from basal T2 values (*P* < 0.05). In the CSA graphs (right), (^∗^) indicates significant differences between PRE and POST CSAs (*P* < 0.05).

**Figure 8** shows the PRE regional T2 shifts and regional CSA changes from PRE to POST. No significant correlations were found between the PRE regional T2 shifts and percentage increase in regional CSAs, except for ST (Total: *r* = -0.043, *P* = 0.164; AM: *r* = 0.131, *P* = 0.142; BFL: *r* = -0.023, *P* = 0.792; BFS: *r* = -0.120, *P* = 0.250; RF: *r* = -0.033, *P* = 0.706; SM: *r* = 0.135, *P* = 0.157; ST: *r* = -0.225, *P* = 0.007; VM: *r* = -0.143, *P* = 0.075; VL+VI: *r* = -0.051, *P* = 0.524).

## Discussion

This study reports significant muscle hypertrophy after only 14 days (five sessions) of a 4 weeks training period. To our knowledge, these findings represent the earliest evidence of macroscopic muscle hypertrophy (without the interference of acute muscle edema) to date.

### Muscle Performance

Fast and notable improvements in muscle performance were seen after the training program (see **Figure 4**). This outcome was favored by the great window of adaptation of the sample (RT naive young volunteers), and the effectiveness of the stimulus applied. The training system used in this study had previously been shown to be more effective in promoting increases in muscle volume, strength and power than conventional weights (Maroto-Izquierdo et al., 2017). The efficacy of the method has been suggested to be mediated by the achievement of higher forces during the ECC phase (Tesch et al., 2004). A marked eccentric overload was not a deliberate outcome when the exercise protocols used in this study were designed, however, force and power values were significantly higher during the eccentric phase (see squat performance results). Similar increases were reported in previous studies involving similar samples and training systems (Seynnes et al., 2007; Fernandez-Gonzalo et al., 2014).

### Muscle Activation

As shown in **Figure 6**, the GM and VL muscles were the most active with the squat exercise. Globally, QUAD muscles increased in T2 intensity, whereas HAMS muscles decreased in intensity. Of the QUAD muscles, however, the activation of RF between participants was highly variable. RF is the bi-articular muscle of the QUAD group, and its activation pattern is highly influenced by the hip position (Miyamoto et al., 2012). Biomechanical differences (i.e., femur lengths) between subjects may have caused different hip flexion angles during execution and thus different RF activation patterns. Concerning HAMS, the T2 shift values reported may appear unexpected, given that previous studies have shown a moderate contribution of hamstrings during squats when assessed by electromyography (Caterisano et al., 2002). It must be noted that T2 values are a quantitative index of the amount and distribution of water in skeletal muscle (Cagnie et al., 2011). When assessed on a single active muscle group, T2 values are highly correlated with electromyographic activity (Adams et al., 1992). However, these two assessment tools have different physiological bases and therefore may lack complete agreement in some situations. When muscles of a whole region (i.e., thighs) are assessed after being non-uniformly recruited, the distribution of water is increased in the most active muscles but it is decreased in less active muscles (Norrbrand et al., 2011). Thus, acute decreases in the T2 signal in HAMS muscles after squat exercise indicate relative inferior activation compared to QUAD muscles, but not a lack of activation (Slater and Hart, 2017).

Regarding the adaptive response to training, no significant changes were found in the T2 shift values from PRE to POST in any of the muscles assessed (**Figure 5**). Therefore, the recruitment pattern did not change over the training period. As exercises for determining T2 shifts were maximal both PRE and POST, it is highly likely that a ceiling was reached over which T2 values did not increase further (Cagnie et al., 2011). However, it is not clear whether the magnitude of the T2 shifts would have been similar if the external load had remained the same in PRE and POST, given that force outputs in the latter were much higher.

### Muscle Hypertrophy

This study shows a rate of increase in QUAD volume of 0.39% per day over the first 14 days of training (5 training sessions), which is almost double what Seynnes et al. (Seynnes et al., 2007) reported (0.2%) for the increase in CSA over the first 20 days of RT (9 training sessions). This difference may be explained by greater exercise volume per session (5 × 10 vs. 4 × 7), as the RT volume seems to be one of the most important factors affecting muscle hypertrophy (Figueiredo et al., 2017). In addition, hypertrophic changes in the study performed by Seynnes et al. (2007) were measured as changes in the CSA of two specific MRI slices at 25 and 50% of the femur bone length. Given that flywheel devices increase tension in the ECC phase of the movement and a distal pattern of hypertrophy has been reported in response to ECC loading (Franchi et al., 2017), changes in the CSAs measured may have underestimated whole-muscle changes. Supporting this idea, data displayed at **Figure 8** show less increase in the middle region of VL+VI (9.9% at 0 cm) and VM (7.6% at 0 cm) compared to their distal regions (VL+VI 21.3% at +9.45 cm; VM 14.0% at +15.75 cm). Given these non-uniform changes, the present study provides a more representative picture of whole-muscle changes by using total muscle volume (Nordez et al., 2009). Lundberg et al. (2013) used a similar flywheel RT volume per session (4 × 7) in combination with intense aerobic exercise, and reported a rate of QUAD hypertrophy (measured as total volume) of 0.4% per day over the first 5 weeks. It should be noted that the rate of CSA volume increase reported in the present study is similar for the first 2 weeks (0.39% per day) but tends to decrease as the trainee progresses (0.30% per day over the first 28 days). The faster rate of increase reported by Lundberg et al. (2013) could have been influenced by the additional training volume of the intense aerobic training, given the untrained condition of the volunteers.

Prior to this study, DeFreitas et al. (2011) reported significant increases in thigh muscle CSA (measured by quantitative computed tomography) after only 1 week (two sessions) of RT. However, testing was performed 48 h after a high-intensity RT protocol performed by unaccustomed subjects. Those authors concluded that early increases in CSA may have been due to edema, and considered that significant skeletal muscle hypertrophy occurred around weeks 3–4. Similarly, Krentz and Farthing (Krentz and Farthing, 2010) also reported significant increases in biceps brachii thickness (measured by ultrasound) after only 8 days (three sessions) of eccentric RT. In that case, the volunteers were tested 48 h after performing a RT session, and there was a concomitant decrease in strength, indirectly indicating that exercise-induced muscle damage was present (Paulsen et al., 2012). Therefore, in both cases, the early increase in muscle size was not considered to be hypertrophy, but edema-induced muscle swelling due to muscle damage (Damas et al., 2016).

It must be made clear that in the present study, the increases in muscle volume were assessed at least 96 h after the last training session to avoid acute muscle swelling. The T2 signal under basal conditions of the muscles assessed did not change significantly from PRE to IN (*P* = 0.101–0.934, with differences of -1.5% ± 1.4%) or from PRE to POST (*P* = 0.137–0.849, with differences of -2.4% ± 1.7%), which indicates the absence of muscle edema (Damas et al., 2016). Additionally, in all cases, pre-RT-session MVIC force levels had recovered when the scans were performed, indirectly indicating the absence of muscle damage (Paulsen et al., 2012). Therefore, we are confident that the changes in muscle volume reported here are accounted for by chronic hypertrophic changes, and not acute processes such as swelling.

### Relationship Between T2 Shift and Hypertrophy

Establishing a relationship between T2 shifts and hypertrophy would be useful as a predictive tool for RT exercises. In fact, strengthening exercises are commonly classified by the magnitude of activation of certain muscles or muscle regions, in order to allow trainers to focus on a specific target of the RT intervention (Fernandez-Gonzalo et al., 2016; Mendez-Villanueva et al., 2016). It must be noted that different approaches are used to assess T2 shifts. For instance, it can be assessed as percentage activated area (Wakahara et al., 2012, 2013), or as percentage change of a representative ROI (Fernandez-Gonzalo et al., 2016; Mendez-Villanueva et al., 2016). Despite both variables commonly being used to quantify the same physiological phenomena, the relationship between them remains unclear. Therefore, differences between procedures may also have influenced the findings discussed here. In any case, the results presented in this study suggest that the regional percentage of change in the T2 signal after exercise is not a reliable tool for predicting the magnitude of increase in CSA, given that no correlation was found between those two variables.

Previous studies have found correlations between the acute T2 shift of specific muscle regions or muscles after exercise and the acute (swelling) (Kubota et al., 2007), and chronic (hypertrophic) (Wakahara et al., 2012, 2013) morphological response measured by MRI. Given this background, a correlation between regional T2 shifts and the percentage of increase in regional CSAs was expected. A plausible explanation for the lack of correlation in the present study is that the previously mentioned studies used highly analytic exercise protocols, in which a given muscle or muscle group worked in isolation. T2 shifts in analytic (i.e., single joint) exercises are related to other variables that are more relevant for promoting muscle hypertrophy (Adams et al., 1992) (such as magnitude and time producing mechanical tension, Schoenfeld, 2010). Although metabolic activity and T2 changes might correlate with mechanical tension and morphological changes in some situations, results suggest that this relation is weakened in complex multi-joint movements such as the squat, in which antagonistic and synergistic muscle groups are recruited throughout the movement (Slater and Hart, 2017). For instance, the HAMS muscle group displays a negative T2 shift in the squat (Norrbrand et al., 2011), despite being electromyographically activated and under tension (Caterisano et al., 2002). As a result, in the present study HAMS muscle volume increased significantly after 4 weeks (see **Table 1**), even though BFL, BFS, SM, and ST showed a significant decrease in the T2 signal after exercise (**Figure 6**).

Therefore, the use of mfMRI as a tool for predicting the potential hypertrophic effects of a given strengthening exercise should be questioned. We encourage future research to consider the physiological basis on which this method relies to correctly interpret mfMRI data from different exercises.

## Conclusion

Muscle hypertrophy is initiated early and is progressive in response to RT, potentially contributing to initial strength gains. In this study, QUAD muscles increased 5.5% ± 1.9% in volume after only 14 days (fivetraining sessions) of flywheel inertial training. This represents the earliest onset of whole-muscle hypertrophy without the interference of acute muscle edema, documented to date. After 4 weeks of inertial squat RT, great increases in knee extensor MVIC (28% ± 17%) and in QUAD muscle volume (8.6% ± 3.6%) were observed.

The application of a robust RT stimulus in combination with a sensitive and precise evaluation tool such as 3D volumetry by MRI has been decisive for these findings. However, this method is at present much more time consuming than the assessment of single CSAs. Therefore, the level of sensitivity and precision needed, and the time available for assessment must be taken into consideration together to decide the best approach in each case (Nordez et al., 2009).

Regional T2 shifts after the first assessment session were not found to be correlated with the relative increase in CSA after the training program. These results call into question the reliability of mfMRI as a tool for predicting the potential hypertrophic outcomes of a given exercise.

## Limitations

In this study, the number of volunteers could be regarded as a limitation on the interpretation of the results. However, it should be taken into account that the responses were very similar in all the participants after the training protocol. Moreover, the sample size of the study was adjusted based on previous related research (Seynnes et al., 2007; Lundberg et al., 2013). Finally, another limitation is the long time currently needed for the volumetric assessment of each muscle. As this precise analysis becomes more automatized with developing imaging technology, research will become easier in the future.

## Author Contributions

VI-D, GC, JP, and JC contributed to the conception and design of the study. VI-D organized the database. VI-D, GC, SN, JP, ML, XA, XP, and JC performed the experiments. VI-D, GC, SN, and JC wrote the first draft of the manuscript. VI-D, SN, GC, JP, XP, ML, RC, XA, and JC contributed to manuscript revision, and also read and approved the submitted version of the manuscript.

## Conflict of Interest Statement

The authors declare that the research was conducted in the absence of any commercial or financial relationships that could be construed as a potential conflict of interest.

## Abbreviations

RT: resistance training
CSA: cross-sectional area
QUAD: quadriceps femoris
CON: concentric
ECC: eccentric
MRI: magnetic resonance imaging
mfMRI: muscle functional magnetic resonance imaging
T2: transverse relaxation time
MVIC: maximal voluntary isometric contraction force
PRE: before the training intervention
IN: after 2 weeks of training intervention
POST: after 4 weeks of training intervention
ROI: region of interest
GM: gluteus maximus
RF: rectus femoris
VI: vastus intermedius
VM: vastus medialis
VL: vastus lateralis
AM: adductor magnus
GR: gracilis
BFL: biceps femoris long head
BFS: biceps femoris short head
ST: semitendinosus
SM: semimembranosus
ADD: adductors
HAMS: hamstrings
